# Investigating Experiences With Scarring Among Transgender and Gender Diverse People: Mixed Methods Study

**DOI:** 10.2196/62714

**Published:** 2025-06-06

**Authors:** Nora Yanyi Sun, Kanika Kamal, Alex Sogomon Keuroghlian

**Affiliations:** 1 Harvard College, Harvard University, 373 Quincy Mail Center 58 Plympton St, Cambridge, MA, 02138, United States, 1 904-646-8255; 2 Harvard Medical School, Cambridge, MA, United States; 3 Department of Dermatology, Brigham and Women’s Hospital, Boston, MA, United States; 4 Department of Psychiatry, Massachusetts General Hospital, Boston, MA, United States; 5 Department of Behavioral Health, Fenway Health, Boston, MA, United States

**Keywords:** transgender, gender-affirming care, scarring, body image, gender, qualitative study, psychosocial, content, online forum, online, physical scarring, thematic analysis, scar, visibility, stress, barrier, marginalized patients, scar visbility, reddit, mastectomy, gender dysphoria, emotional trauma, psychosocial experience, trauma

## Abstract

**Background:**

Scarring has been shown to have adverse health effects on marginalized patient groups. However, experiences of scarring among transgender and gender diverse (TGD) people have not yet been thoroughly characterized.

**Objective:**

This study aimed to investigate the impacts of scarring related to gender-affirming care and other causes among TGD people.

**Methods:**

Anonymous data were extracted from Reddit, a popular online platform organized into “subreddit” groups based on identities and interests. A combined total of 604 posts and comments that explicitly reference physical scarring were extracted from r/FtM, a subreddit for transmasculine people (449 posts and comments) and r/MtF, a subreddit for transfeminine people (155 posts and comments). Applying inductive thematic analysis, all posts and comments were coded and codes were sorted into overarching themes.** **

**Results:**

Among the 604 posts and comments, the scars most discussed were secondary to gender-affirming care procedures, including mastectomy (n=338 posts and comments), hormone administration (n=102 posts and comments), and hair removal (n=38 posts and comments). Nongender-affirming care-related scars, such as those due to self-harm (n=43 posts and comments), were discussed less often. A total of five overarching themes emerged through thematic analysis: (1) concerns about physical outcomes related to scarring; (2) psychological distress related to scarring; (3) societal perceptions of scarring; (4) strategies to prevent, conceal, and minimize scarring; and (5) positive experiences with scarring.

**Conclusions:**

For TGD people, scar complications, visibility, and permanence represent major concerns. While many TGD people ultimately accept scarring as an unavoidable consequence, scarring both related and unrelated to gender-affirming care can present a significant psychosocial stressor for TGD people. Scarring can result in physical health complications, gender dysphoria, and negative body image; visible scarring is also a barrier for TGD people who wish to blend into society. Clinicians should improve communication regarding scarring outcomes and scar-care procedures. Future research should focus on the development of scar prevention, care, and reduction techniques for TGD people.

## Introduction

“Transgender and gender diverse” (TGD) is an umbrella term for people whose gender identities differ from societal expectations related to the sex they were assigned at birth. Some TGD people may choose to undergo gender-affirming surgery or pursue other forms of gender-affirming care at any point in their lives to alleviate gender dysphoria and better align their physical characteristics with their gender identity. Gender-affirming care is considered medically necessary and associated with numerous mental health benefits, including significantly decreased psychological distress and suicidality [1].

Over the past 2 decades, the number of gender-affirming surgeries among TGD people has increased. Approximately 9000 gender-affirming surgeries now occur annually across the United States [1,2], thus likely resulting in an increased prevalence of surgical scarring. Although appearance and permanence vary broadly by surgical techniques [3], scarring remains a concern for some TGD people.

In addition to gender-affirming surgery, gender-affirming care more broadly, including nonsurgical interventions such as estrogen and testosterone injections and patches, may also result in significant scarring when used for long-term therapy [4]. In addition, gender-affirming hormones may cause increased acne scarring [5,6]. Furthermore, gender-affirming hormones may lead to greater visibility of pre-existing scars and predisposition to scarring due to changes in skin composition [7]. Similarly, hair removal procedures may result in scarring if not performed properly [8]. Finally, due to the unique combination of stressors experienced by TGD populations, higher rates of self-injury, a coping mechanism among some TGD people, may also be a cause of scarring [9].

Scarring may reveal TGD identities to other people and thereby interfere with goals of blending into society, thus potentially resulting in significant stress for TGD people [3]. Furthermore, studies have shown that scarring may cause significant mental health burden, especially in marginalized and minoritized populations; for example, scarring is associated with adverse psychological outcomes among breast cancer survivors and people of color [3]. Within minority stress theory, minoritized populations face a variety of external and internal stressors. As scarring is often perceived as a negative physical trait by society, bearing visible scars is an additional form of marginalization, and multiply marginalized people may experience superadditive stressors, exclusion, and discrimination, explaining the increased psychological distress observed among marginalized people with scars [10].

Furthermore, TGD people are uniquely vulnerable to body dissatisfaction because they experience gender dysphoria and increased body scrutiny from others. Per body objectification theory, increased awareness of one’s body, especially physical characteristics that do not align with personal or societal goals, may lead to body shame, which is linked to increased psychological distress and disordered eating [11]. Thus, compared with cisgender people, TGD people may be especially vulnerable to body dissatisfaction due to their scars.

The impacts of scars on TGD people have not been well-characterized in the literature. Currently, most studies investigate scarring among TGD people through a surgical and dermatologic rather than psychological and societal lens [12]. Also, most studies have limited their scope to scarring directly caused by traditionally defined gender-affirming surgery [13]. Consistent with objectification theory, these limited studies with TGD populations have suggested that scarring may lead to gender dysphoria, body image dissatisfaction, and symptoms of both depression and anxiety (eg, breast augmentation surgery scars may reduce self-esteem or lead to negative self-perception) [14].

Although some studies mention scarring due to nonsurgical gender-affirming practices, such as chest binding, these studies focus on the overall impacts of these gender-affirming practices rather than specifically centering the impacts of scars on TGD people [15].

As physical appearance plays an important role in body image and self-perception [16], scarring that is not explicitly connected with gender-affirming care may represent a substantial portion of TGD people’s scars and has the potential to affect them in unique ways that warrant study.

Understanding TGD people’s experiences with scarring is necessary to motivate and inform innovation in scar treatments and supportive psychological care. Furthermore, treatment of scarring related or unrelated to gender-affirming care is currently deemed cosmetic and not medically necessary by most insurance companies; it is therefore not covered under most insurance policies [3]. An improved understanding of the impact of scarring for TGD people could support the medical and psychological necessity of scar treatment, and potentially more inclusive revisions to these policies.

Reddit is a popular online platform where users can enter long-form posts, comment on posts, and respond to comments. Due to the anonymous nature of the platform, it attracts a large number of TGD people to discuss personal health matters. Reddit forums have been studied to glean a better understanding of mental health [17,18] and sexual health [19], as well as LGBT (lesbian, gay, bisexual, and transgender) health [20]. The Reddit website is organized into subreddits centered around a specific subject, connecting people with similar identities or interests in dialogue.

“r/MtF” (“male-to-female”) and “r/FtM” (“female-to-male”) communities on Reddit are a pair of online subreddits for TGD people that host approximately 470,000 total users. In this study, we investigated the experiences of physical scarring among TGD people by analyzing data extracted from these 2 online forums and characterized the common experiences of scarring in these communities by identifying 6 common themes of the threads.

We note that the subreddits names “MtF” and “FtM” are based on an outdated, binary conception of gender [21] and inappropriately emphasize the sex assigned at birth. Therefore, this study may fail to adequately represent the experiences of TGD people with scarring, including nonbinary people, as well as experiences of TGD people who do not feel comfortable using these subreddits. From here onwards, this paper will refer to r/MtF as the transfeminine subreddit and r/FtM as the transmasculine subreddit.

## Methods

### Data Collection

Threads–defined as posts plus any comments on those posts–in the transfeminine and transmasculine community subreddits were screened by entering the keyword “scar” in the subreddit search function to extract threads that contain this term, including alternative forms of the word, such as “scarring.” This method has been used in previous studies analyzing Reddit data [17].

As Reddit’s search feature caps the number of threads that can be returned at a given time, we initially manually downloaded the 70 most recent qualifying threads from each subreddit on December 21, 2023. Threads that did not mention scarring or only mentioned scars in a metaphorical context were marked for removal from the dataset. Following removal of posts and comments that did not meet the study’s search criteria, a total of 449 posts and comments from the transmasculine subreddit and 155 posts and comments from the transfeminine subreddit were coded.

Based on the concept of information power, an alternative to data saturation, no further threads were downloaded after the initially extracted posts because analyzed threads were information-dense, sometimes with hundreds of relevant comments; Furthermore, the study had a focused goal to examine effects of scarring among TGD people that was fulfilled by the initial analysis [22,23].

### Data Analysis

We performed inductive thematic analysis, as described by Braun and Clarke [24], by applying a nonpositivist “big Q” approach that acknowledged reflexivity and aimed at interpreting the qualitative data. The study team consisted of 3 authors, including 2 cisgender women and a nonbinary person; all members of the study team also identified as queer, a person of color, or both.

Before coding, each post and comment included in the analysis was assigned a row in a spreadsheet. Codes were then assigned to each row in this spreadsheet. Two authors then independently coded the first 14 posts and related comments in each dataset on their own copy of the spreadsheet, at which point both authors believed that they understood the data sufficiently to develop an initial codebook. The codes were then reviewed jointly by both authors to remove duplicated codes and combine the rest of the codes until consensus was reached on a working codebook. During the discussion process, the coders used reflexivity practices, such as reflecting on potential personal biases and verbalizing any assumptions made during coding.

The remainder of the threads was then coded by 1 author using the working codebook, amending the codebook as necessary. Coding also involved noting the type of scar in each post and comments to contextualize the results. The senior author provided clinical perspectives and critical suggestions throughout coding. Through discussion with the senior author, the coders organized the codes into a set of 6 overarching themes.

### Ethical Considerations

The transfeminine and transmasculine subreddits, including all threads analyzed, are located within the public domain. On the Reddit platform, users are identified only by usernames, which are generally not connected to any identifying information. During the coding process as well as in this article, all user data were completely deidentified, with no usernames recorded. This study received a “Not Human Subjects Research Determination” from the Harvard Longwood Area Institutional Review Board because the authors are not able to ascertain the identities of the users whose posts are included in the analysis. To further preserve user anonymity, quantitative data are only presented in aggregated form, and we have conducted slight and judicious rephrasing of quotes while preserving meaning to prevent the original posts from being retrieved [25].

## Results

### Types of Scarring

Among posts and comments in the transmasculine subreddit, mastectomy scars were highly represented, comprising 75.3% (338/449) of posts and comments. Second to mastectomy scars were scars due to testosterone injection, comprising 17.4% (78/449) of posts and comments. These 2 types of scars represented a combined total of 92.6% (416/449) of all posts and comments in the transmasculine subreddit (Table 1).

**Table 1.** Types of scars in the transmasculine subreddit.

Type of scars	Count (n=449), n (%)	
Mastectomy scars	338 (75.3)	
Scarring due to testosterone administration	78 (17.4)	
Self-harm scars	5 (1.1)	
Other scars unrelated to gender-affirming care	15 (3.3)	
Other scars related to gender-affirming care	8 (1.8)	

In comparison with the transmasculine subreddit, sources of scars discussed on the transfeminine subreddit were more divergent. Self-harm scars (38 posts and comments, 24.5%), scarring due to hair removal procedures (29 posts and comments, 18.7%), and scarring due to estrogen administration (24 posts and comments, 15.5%) were the most common types of scars discussed. Scarring that is directly attributed to gender-affirming procedures comprised a majority of the threads (86/155, 55.5%), with a broad variety of both surgical and nonsurgical procedures mentioned, including hair removal, vaginoplasty, breast augmentation, facial feminization surgery, orchiectomy, and genital tucking (Table 2).

**Table 2.** Types of scars in the transfeminine subreddit.

Scar type	Count (n=155), n (%)	
Self-harm scars	38 (24.5)	
Scarring due to hair removal procedures	29 (18.7)	
Scarring due to estrogen administration (eg, patches and injection)	24 (15.5)	
Orchiectomy scars	11 (7.1)	
Vaginoplasty scars	9 (5.8)	
Acne scars	8 (5.2)	
Stretch marks	6 (3.9)	
Other scars related to gender-affirming care	13 (8.4)	
Other scars unrelated to gender-affirming care	17 (10.9)	

### Qualitative Themes

Five overarching themes describing various experiences with scarring emerged through inductive thematic analysis: (1) concerns about physical outcomes related to scarring; (2) psychological distress related to scarring; (3) societal perceptions of scarring; (4) strategies to prevent, conceal, and minimize scarring; and (5) positive experiences with scarring. All 5 themes were identified in both subreddits and both in and beyond the context of gender-affirming care procedures (Table 3).

**Table 3.** Frequencies of codes and themes identified.

Themes and subthemes	r/FtM^a^ (n=551), n (%)	r/MtF^b^ (n=165), n (%)
Theme 1: concerns about physical outcomes related to scarring	83 (14.4)	62 (35.8)
Interference of pre-existing scars with gender-affirming care	6 (1)	19 (11)
Desire for specific scar appearance	20 (3.5)	7 (4.1)
Period of decreased mobility due to scarring	15 (2.6)	0 (0)
Scar sensitivity	7 (1.2)	2 (1.2)
Scar tissue build-up due to nonsurgical gender-affirming care	7 (1.2)	9 (5.2)
Hormone absorption on scars	13 (2.2)	0 (0)
Heterogeneity in scar healing	9 (1.5)	1 (0.6)
Scar evolution	2 (0.3)	12 (6.9)
Encouraging communication with physician regarding physical health concerns related to scarring	4 (0.7)	12 (6.9)
Theme 2: psychological distress related to scarring	19 (3.3)	28 (16.2)
Anxiety about increased predisposition to scarring	1 (0.2)	5 (2.9)
Anxiety about possible scar appearance	6 (1.0)	9 (5.2)
Hesitation to pursue gender-affirming care due to worries regarding scar permanence	5 (0.8)	3 (1.7)
Negative body image due to scarring	7 (1.2)	6 (3.4)
Scars as source of gender dysphoria	0 (0)	5 (2.8)
Theme 3: societal perceptions of scarring	131 (22.8)	2 (1.2)
Fear of identity revelation due to gender-affirming care scars	20 (3.5)	2 (1.2)
Transphobic views of scarring as mutilation	14 (2.4)	0 (0)
Exaggerated representations of scarring	97 (16.8)	0 (0)
Theme 4: strategies to prevent, conceal, and minimize scarring	260 (45.2)	62 (35.8)
Inquiry for scar prevention advice	6 (1.0)	0 (0)
Sharing scar prevention experience	9 (1.5)	11 (6.3)
Inquiry for scar care advice	6 (1.0)	3 (1.7)
Uncertainty about scar care due to poor communication from gender-affirming care clinician	27 (4.7)	3 (1.7)
Sharing scar care experiences	24 (4.2)	0 (0)
Scar concealment with clothing	31 (5.4)	3 (1.7)
Tattooing scars	5 (0.8)	1 (0.6)
Lasering scars	19 (3.3)	2 (1.1)
Concealing gender-affirming surgery scar origin	72 (12.5)	33 (19.1)
Scar revision surgery	55 (9.6)	5 (2.9)
Encouraging communication with physician regarding preventing, concealing, and minimizing scarring	6 (1.0)	1 (0.5)
Theme 5: positive experiences with scarring	82 (14.3)	19 (10.9)
Preference for gender-affirming care despite scarring	57 (9.9)	1 (0.5)
Low visibility of scars	1 (0.2)	10 (5.7)
Scar improvement over time	9 (1.57)	0 (0)
Acceptance of and pride in scarring	15 (2.61)	8 (4.6)

a r/FtM: transmasculine subreddit.

b r/MtF: transfeminine subreddit.

### Theme 1: Concerns About Physical Outcomes Related to Scarring

Posts discussed physical health concerns related to existing or future scars that were both related and unrelated to gender-affirming care (Table 4). Posts discussed the physical health implications of scarring, such as how to manage scarring-related complications, what the expected course of recovery and outcomes of scarring are, and whether scarring would make them ineligible for certain gender-affirming procedures. Many asked for advice on how to treat scars following gender-affirming surgery due to lack of effective communication from their care teams, which sometimes resulted in divergent responses. Posts shared concerns regarding the impact of pre-existing scars on eligibility, feasibility, and effectiveness of gender-affirming care. Posts also expressed concern that scarring near treatment sites for gender-affirming surgery or hormone therapies may render the treatment not physically feasible or effective for them. Posts also described users’ experiences of and asked for advice regarding severe scarring due to improper technique administering gender-affirming hormones with injections or patches.

**Table 4.** Concerns about physical outcomes related to scarring.

Sample codes	Selected quotes
Desire for specific scar appearance	*The shape I prefer for my own operation is scars straight across. *[Transmasculine subreddit, mastectomy scars]
Period of decreased mobility due to scarring	*I couldn’t stay after returning to my old job because even after 8 weeks I couldn’t handle the heavy lifting. I still can’t do it. I am unemployed right now and the scars are making it harder to find a job.* [Transmasculine subreddit, mastectomy scars]
	*Don’t move your arms or carry heavy objects for several weeks or months because this can stretch out the scars. *[Transmasculine subreddit, mastectomy scars]
Scar tissue build-up due to nonsurgical gender-affirming care (eg, gender-affirming hormones patches and gender-affirming hormones injections)	*I’ve been using hormone patches for about 5‐6 months now. I always shave and clean the area before application, but no matter how I take off the patches, it causes bleeding and scarring. I don’t know what to do. I’m almost out of safe areas to apply the patches because of the scar tissue.* [Transfeminine subreddit, scarring due to estrogen administration]
Interference of pre-existing scars with gender-affirming care	*Will having scars on my chest make it hard for me to get top surgery in the future? I have some scars on my chest below my collarbones. These scars aren’t severe, but I’m worried they could cause problems.* [Transmasculine subreddit, mastectomy scars]
	*I have many scars on the front of my thighs, which are not wounds. Would this affect testosterone absorption at all?* [Transmasculine subreddit, unspecified scars unrelated to gender-affirming care]

### Theme 2: Psychological Distress Related to Scarring

Before receiving gender-affirming care, posts expressed anxiety regarding the appearance, visibility, and permanence of scars that may result from these procedures (Table 5). Specifically, users expressed anxiety about uncertainty and heterogeneity in scarring outcomes and changes in scar appearance over time, and some posts expressed that users had increased desire for visible scarring. Posts sometimes specified that concern and confusion about scarring outcomes had resulted from poor communication from a gender-affirming surgeon or other clinician.

**Table 5.** Psychological distress related to scarring.

Sample codes	Selected quotes
Anxiety about possible scar appearance	*But I started thinking, what if the surgery pulls at my skin or something else goes wrong and I get some outer scar?* [Transfeminine subreddit, vaginoplasty scars]
	*I am currently waiting for my top surgery to be scheduled. My physician said my scar will be a little rounded with the cuts. I am worried that the scar might be too rounded, and I won’t like the appearance.* [Transmasculine subreddit, mastectomy scars]
	*I wanted to know about scar placement and nipple placement, if he’s worried about dog ears or if I might need revision later, but he talks so fast I could barely get a word in or process everything he was telling me.* [Transmasculine subreddit, mastectomy scars]
Negative body image due to scarring	*I hate almost everything about my body: My face is too masculine and I hate the scars I’ve given myself.* [Transfeminine subreddit, self-harm scars]
	*I think I have the unluckiest batch of genes yet… 7’8,” looks like Kaiser Wilhelm II, and a boxy face with scars.* [Transfeminine subreddit, unspecified scars unrelated to gender-affirming care]
	*I love showers, so dreading them because I have to see my scars only made me more upset.* [Transmasculine subreddit, mastectomy scars]

### Theme 3: Societal Perceptions of Scarring

When exchanging advice regarding scar care and concealment, many posts mentioned users’ motivations for altering scar appearance to improve social acceptance, representing the third theme of the threads (Table 6). While many motivations were internal, like those mentioned in the previous theme, some posts described motivations related to societal perceptions of scarring. Specifically, posts shared concerns about being “clocked”—recognized as TGD—due to prominent surgery scars. Some posts also expressed concerns about receiving transphobic comments that characterize their gender-affirming surgery scars as evidence of self-mutilation, as well as general aversion among family, friends, and community members toward scars of any origin.

**Table 6.** Societal perceptions of scarring.

Sample codes	Exemplary quotes
Fear of identity revelation to other people due to gender-affirming care scars	*Does anyone have any tips about what I could say if someone clocked the scars on my vagina or clocked me in general? Even though I believe I’m stealth, I still worry a lot that I’ll get clocked wherever I go.* [Transfeminine subreddit, vaginoplasty scars]
	*I am having top surgery soon, and I’m super excited for it, but I’m really not looking forward to having visible scars. I don’t want to get clocked by people, especially with trans scars becoming more recognized.* [Transmasculine subreddit, mastectomy scars]
Transphobic views of scarring as mutilation	*My mother says I’m paying thousands of dollars to mutilate myself.* [Transmasculine subreddit, mastectomy scars]
	*My entire family is vehemently against “mutilating” a healthy body part.* [Transmasculine subreddit, mastectomy scars]

### Theme 4: Strategies to Prevent, Conceal, and Minimize Scars

Users made posts and comments exchanging advice on topics surrounding changing the appearance of scars—both related and unrelated to gender-affirming care, which represents the fourth theme of the threads (Table 7). Oftentimes, posts shared and asked for advice on scar care techniques that reduced and concealed scarring. Specifically, many users on the transmasculine subreddit exchanged advice for mastectomy scar aftercare so that the healed scar lines would be aesthetically pleasing or minimally visible. Various posts also discussed ways to conceal scarring. Popular methods for concealing a variety of scars included tattoos (especially over mastectomy scars), make-up, body hair, clothing, and laser treatments for scar revision.

**Table 7.** Strategies to prevent, conceal, and minimize scars.

Sample codes	Exemplary quotes
Sharing scar prevention experience and advice	*I was advised to wear a binder or ace bandages to help keep the chest shape in line.* [Transmasculine subreddit, mastectomy scars]
	*After surgery, do not carry heavy objects for several weeks or months as that can stretch out the scars.* [Transmasculine subreddit, mastectomy scars]
Scar concealment with clothing	*I have a scar on my chest that I hate showing off. Are there any bras out there that cover up the sternum?* [Transfeminine subreddit, other scars related to gender-affirming care]
Tattooing scars	*Does anyone know how long you should wait until you can tattoo your top surgery scars? If anyone has tattoos on their scars, how was it? Any advice would be great, thank you.* [Transmasculine subreddit, mastectomy scars]
	*I lost a lot of definition and pigment during healing, and I think I would be happier with my nipples if they were enhanced by a tattoo artist.* [Transmasculine subreddit, mastectomy scars]
Concealing gender-affirming care scar origin	*If they ask what your scars are, you could say you had gynecomastia, which is where a biological male grows breast tissue due to a hormone imbalance. You could say you had weight loss surgery.* [Transmasculine subreddit, mastectomy scars]

### Theme 5: Positive Experiences Related to Scarring

Despite expressing negative views about scarring, posts communicated a strong preference for scarring over the alternative of not receiving gender-affirming care (Table 8). Although many posts expressed frustration at the failure of modern surgical and dermatologic techniques to eliminate scarring, no posts in the sample describe regretting receiving gender-affirming care. Some posts reported satisfaction with scar appearance and even expressed acceptance of and pride in scars. A few posts in the transmasculine subreddit described associating scarring with masculinity and finding scars to be a source of gender euphoria. Some users shared that they viewed scarring as a valuable part of the TGD experience or described scarring as a source of bonding over shared experience within TGD communities.

**Table 8.** Positive experiences related to scarring.

Sample codes	Selected quotes
Preference for gender-affirming care despite scar visibility	*Obviously, the presence of scars is preferable to having dysphoria.* [Transmasculine subreddit, mastectomy scars]
	*I know I am very privileged to be able to get top surgery, and I would definitely rather have scars than not have surgery.* [Transmasculine subreddit, mastectomy scars]
Scar improvement over time	*Just know that most scars fade really well, especially after 3+ years.* [Transmasculine subreddit, mastectomy scars]
	*My skin has come back from some pretty bad times with scarring before and even though I still relapse a bit it does give me hope that my skin is capable of clearing up again eventually.* [Transmasculine subreddit, other scars unrelated to gender-affirming care]
Acceptance of and pride in scarring	*I refuse to be ashamed of my suicide scars, and I believe you’ll have the power to dismiss the bad feelings about your scars one day, too.* [Transfeminine subreddit, scarring due to suicide attempt]
	*I love the connection I feel with other trans people, especially other transmascs, when we compare surgical scars, share resources, and share old garments or pillows that we don’t need anymore.* [Transmasculine subreddit, other scars related to gender-affirming care]

## Discussion

### Principal Findings

TGD people are at increased risk of having scars from a variety of causes, both related and unrelated to gender-affirming care. Some TGD people see their scars as a physical representation of their resilience and authentic journey, while for other TGD people, scarring presents a psychosocial burden.

This study shows that scars may generate stress and anxiety for TGD people in various ways. Most directly, many TGD people experience stressful physical health concerns related to scarring. These include but are not limited to (1) how to properly care for scars; (2) how to administer gender-affirming interventions, such as gender-affirming hormones or hair removal, in a manner that minimizes injury and scarring; and (3) whether scarring and associated injuries may render certain gender-affirming procedures infeasible for them to pursue.

The prevalence of these concerns highlights a need for safer, standardized scar care procedures. A variety of scar care strategies, many of which have not been clinically validated and some of which may not be safe or effective, were exchanged on the subreddit. Furthermore, posts expressed frustration and stress regarding the divergent nature of recommendations that users obtained on the internet, from peers, and from their care teams.

In addition, clinicians should fully and clearly address potential physical health concerns related to scarring, such as how to administer gender-affirming interventions in a safe manner that minimizes scarring, and how pre-existing scars may impact eligibility for subsequent gender-affirming treatments. Beyond creating psychological distress for TGD people, suboptimal communication with care teams may contribute to adverse physical consequences, as some posts reported experiences of severe scar tissue build-up and scar infection.

Aside from physical health concerns, posts also shared concerns about scar shape and visibility. These concerns stemmed from both internalized sources, such as negative body image and gender dysphoria due to scarring, and external sources, such as transphobic reactions from other people against physical scars [26].

In a number of instances, posts attributed these concerns to suboptimal communication with the TGD person’s care team. In particular, posts shared experiences of clinicians providing inadequate information on scarring outcomes and scar care procedures. Users also reported not having the opportunity or the comfort to ask questions. Specific obstacles to effective communication with care teams included clinicians who spoke too quickly or unclearly, who were not accessible outside of appointments, and who were dismissive of concerns. These findings are consistent with published literature that found that the providers who were most positively reviewed by TGD patients had in-depth clinical knowledge of gender-affirming care [27], which is demonstrated at least in part by adequately explaining interventions and addressing any patient concerns.

Several posts described anxiety due to uncertainty about how to deal with scarring and interest in hearing peer advice about gender-affirming procedure selection and scar minimization strategies. In addition to providing accounts of personal experiences, commenters on these posts frequently offered words of encouragement and support. Interactions on the subreddits, despite the large number of posts and anonymity, were almost always positive. These findings are consistent with previous literature characterizing how the lived experience of peers is key in facilitating decision-making and providing social support for TGD people undergoing gender-affirming care [28].

While the majority of posts mentioned the impact of scarring on self-perception, some posts also cited external stressors, such as stigma against scars and gender-affirming care scars as a potential obstacle to identity concealment. Several posts expressed that the permanence and visibility of scarring was a deterrent to pursuing gender-affirming care. Other posts stated that family members or friends refused to support pursuing gender-affirming care due to the scarring that the treatments might cause, specifically mentioning transphobic comments that associated scarring from gender-affirming surgery with “mutilation.”

Importantly, potential negative psychosocial consequences of scarring from gender-affirming care are not a valid argument against providing gender-affirming interventions. Many posts explicitly prefaced their negative sentiments toward scarring with a strong desire for receiving gender-affirming interventions over not having the scars. Posts primarily described frustration at the limitations of current treatment techniques in minimizing scarring, and sometimes also at the lack or the cost of scar-minimizing treatments. The negative psychosocial impacts of scarring documented in this study highlight the necessity for continued improvement in scar-minimizing procedures.

Finally, several posts did not share negative views of scarring, described satisfaction with scar outcomes, or expressed finding their scars to be either a source of gender euphoria or a way to bond with other TGD people. These findings suggest that preferences for scar visibility and appearance are heterogeneous and influenced by a complex interplay of psychosocial factors, similar to TGD people’s diverse desires and priorities with regard to different types of gender-affirming interventions [29].

### Limitations

This study is limited by a Reddit-based sample of experiences that may not be representative of the experiences of all TGD people. Reddit users are predominantly white young adults based in the United States [30]. Furthermore, certain themes may be overrepresented or underrepresented as a consequence of the Reddit algorithm, which may boost certain types of threads to the forefront of users’ pages and thus result in these topics receiving disproportionate attention within the sample of threads analyzed in this study.

The majority of posts analyzed in this study expressed negative or neutral experiences with scarring. A growing body of literature suggests that a significant portion of TGD people may take pride in their scars and even derive gender euphoria from them [31]. Although it has been well-established that TGD people have diverse body goals, many of which may not involve passing as cisgender, these sentiments were underexpressed in the data analyzed in this study, potentially because people who have negative experiences related to scarring are more likely to seek out the subreddit to vent or ask for advice. Thus, the prevalence results should be considered in the context of the limitations of analyzing organically occurring data.

Overall, this exploratory study suggests that there can be significant negative psychological impacts associated with scarring from various sources among TGD people. Future research ought to focus on further analyzing psychosocial experiences associated with specific types of scarring mentioned in this study in order to gain greater depth of understanding and develop effective scar treatment options.

### Conclusions

TGD people on Reddit describe a wide variety of experiences with scarring. TGD people discussed scars secondary to gender-affirming care interventions, such as mastectomy and hair removal, and scarring not explicitly related to gender-affirming care, as in instances of self-harm and acne. Some TGD people expressed acceptance of and appreciation for their scars; for other TGD people, scarring represented a significant source of psychosocial stress. Internally, scarring may trigger complex feelings of gender dysphoria and trauma; externally, scarring may present an obstacle to identity concealment and render TGD people vulnerable to stigmatization and transphobic hostility.

These concerns underscore the need for improvement of scar minimization procedures and standardization of safer and culturally responsive scar care. Reddit threads also highlight a need for clearer and more thorough communication from gender-affirming care teams, as well as the potential positive impact of TGD peers in facilitating decision-making and providing social support. Future studies should analyze the psychosocial impacts of specific types of scarring among TGD people with greater depth to better inform medical innovation and health care policy.

## Abbreviations

LGBT: lesbian, gay, bisexual, and transgender
r/FtM: transmasculine subreddit
r/MtF: transfeminine subreddit
TGD: transgender and gender diverse
